# Design of a Single-Layer High-Efficiency Ultra-Wideband Polarization-Converting Metasurface

**DOI:** 10.3390/mi17050576

**Published:** 2026-05-07

**Authors:** Qilin Ren, Shuang Ma, Jiahao Liu, Ya Fan, Ying Yu, Huilin Mu, Sihang Tian

**Affiliations:** 1 School of Electronic Information Engineering, Shenyang Aerospace University, Shenyang 110034, China; renqilin@stu.sau.edu.cn (Q.R.); liujiahao8@stu.sau.edu.cn (J.L.); 2 Air Force Early Warning Academy, Wuhan 430019, China; 3 Chinese Academy of Military Science, Beijing 100091, China; yuying080222@163.com; 4 Air Force Engineering University, Xi’an 710051, China; muhuilin@alu.hit.edu.cn (H.M.); 19029155656@163.com (S.T.)

**Keywords:** polarization conversion, metasurfaces, ultra-wideband

## Abstract

In this paper, we propose a single-layer metasurface structure with ultra-wideband operation and high polarization conversion efficiency, capable of transforming linearly polarized waves into cross-polarized waves. This structure excites additional electromagnetic resonance modes by integrating two symmetrical square patches within an anisotropic split-ring resonator (SRR). These new modes couple with the inherent resonance modes of the SRR, forming closely spaced multi-resonance characteristics across a wide frequency band. This multi-resonance capability enables broadband polarization conversion. This metasurface achieves an ultra-wideband performance spanning 10.89 GHz to 30.12 GHz, covering part of the X-band, the entire Ku-band, and the K-band, while maintaining a high polarization conversion efficiency exceeding 90%. Its broadband characteristics are attributed to the resonator’s ability to generate multiple resonances within a single unit cell. Both experimental and simulation results demonstrate the metasurface’s excellent polarization conversion performance. Furthermore, the proposed metasurface maintains acceptable oblique-incidence performance over a large portion of the operating band, although localized degradation appears at some frequencies. This structure offers significant advantages over traditional multilayer or active designs, featuring simple fabrication without assembly or welding. It may be useful for broadband polarization conversion and may also provide potential for scattering-control applications.

## 1. Introduction

In recent years, artificial metamaterials and metasurfaces have achieved significant breakthroughs in wavefront shaping, absorption, filtering, and polarization control due to their precise manipulation of electromagnetic wave propagation. As the two-dimensional counterpart to metamaterials, metasurfaces achieve electromagnetic response characteristics unattainable by natural materials by designing unit structures at scales far smaller than the wavelength. This enables novel and compact solutions for microwave communications [1], radar systems [2], biological sensors [3], and RCS (radar cross section) reduction [4]. Among these, polarization-converting metasurfaces [5] can effectively transform the polarization state of an incident wave into a target polarization form. This capability is crucial for enhancing the anti-interference capability of communication links, enabling polarization multiplexing, and boosting sensor sensitivity.

To achieve more practical polarization conversion functionality, researchers typically focus on broadening the device’s operating bandwidth or enabling multi-band operation. To this end, structures such as combinations of multiple resonant units, complex symmetry designs, or multilayer stacking are commonly employed to broaden the bandwidth. For instance, Reference [6] proposes an efficient broadband dual-V-shaped polarization-converting metasurface. Reference [7] achieves efficient polarization conversion by designing a double-ring metasurface. Reference [8] proposes a quadruple L-shaped structure to realize efficient broadband polarization conversion. Additionally, numerous other types of metasurfaces for broadband polarization conversion exist, such as H-shaped structures [9], square split-ring resonators (SRRs) [10], dual-W configurations [11], and interembedded lock-shaped linear polarization converters [12].

Recent studies show that researchers are no longer focusing only on bandwidth enhancement, but are also exploring multifunctional and reconfigurable polarization-converting metasurfaces. Zhou et al. [13] proposed a reflective metasurface with dual-broadband and dual-function polarization conversion, while Zhao et al. [14] introduced active elements to realize tunable multifunctional responses. In a related direction, Gao et al. [15] used a vanadium-dioxide-based THz metasurface to switch between wideband absorption and polarization conversion. Together, these studies show that extending device functionality has become an important direction alongside broadband performance. Meanwhile, recent studies have further extended metasurface research to related directions such as holographic imaging, spatiotemporal encoding, anomalous reflection control, multibeam antenna design, and directional scattering enhancement [16,17,18,19,20], which reflects the broad and rapidly expanding application scope of metasurface-based electromagnetic manipulation.

Another important route toward broadband polarization conversion is to employ multilayer structures, which can effectively introduce additional resonant modes and broaden the operating bandwidth. For instance, Li et al. [21] designed a three-layer metasurface that successfully achieved broadband polarization conversion. Reference [22] employed a three-layer stacked rotated split ring to realize multi-mode and multi-band polarization conversion. Furthermore, studies have reported dual-layer polarization-converting metasurfaces capable of stable operation across wide frequency bands [23,24,25]. Recently, introducing air gaps between layers in multilayer designs has further enhanced operating bandwidth [26,27]. Currently, polarization-converting metasurfaces based on air gap structures typically rely on post-assembly, leading to complex manufacturing processes, thick overall structures, and challenges in controlling assembly precision. This hinders large-scale, low-cost practical applications. Although broadband conversion performance has been achieved in previous studies [28,29,30], multilayer designs often come at the cost of increased structural thickness and manufacturing difficulties. Therefore, how to achieve easy-to-process designs through structural simplification while maintaining efficient broadband performance remains a key research topic in this field.

To address the limitations noted above, we designed a single-layer passive polarization-converting metasurface based on a nested multi-resonator topology. By tuning the key geometric parameters, we obtained four closely spaced resonance dips at 11.91, 18.20, 26.29, and 29.77 GHz, which together form a continuous and broad polarization conversion band. Experimentally and numerically, the proposed metasurface achieves a polarization conversion ratio (PCR) higher than 90% over the 10.89–30.12 GHz band, covering the X, Ku, and K bands, while maintaining a low-profile and fabrication-friendly structure that is compatible with standard printed-circuit-board processes and does not require soldering or post-assembly steps.

We also carried out a systematic parametric study to examine how the key dimensions affect the position, depth, and width of each resonance. By analyzing the phase responses, surface current distributions, and parametric results, we found that the broadband behavior originates from the interaction of multiple adjacent resonant modes. Compared with previously reported designs, the proposed metasurface provides a more favorable balance among continuous ultra-wideband operation, high polarization conversion efficiency, low profile, acceptable angular stability, and low fabrication complexity. These features further highlight the practical value of the proposed design in scenarios where both broadband performance and structural simplicity are required.

Section 2 focuses on the design of the proposed metasurface and its simulated performance. We then explain the operating principle from the perspectives of phase response, surface current distribution, and parametric analysis, and further discuss the angular stability and scattering-related application potential. Section 3 gives the fabrication details, measurement results, and the corresponding error analysis. Section 4 concludes the paper.

## 2. Materials and Methods

### 2.1. Structural Design

**Figure 1:**
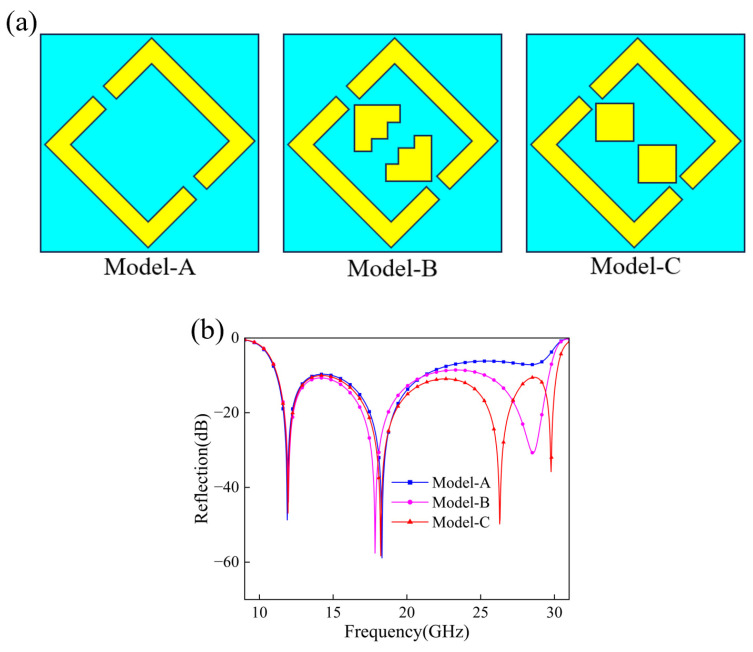
(**a**) Schematic Diagrams of Different Cell Types. (**b**) Co-polarization Reflection Coefficients.

Model-A adopts a square split-ring resonator as the basic structure, because this type of resonator can provide the anisotropic electromagnetic response required for reflective polarization conversion. As shown in Figure 1a, Model-A produces two resonance points near 12 GHz and 18 GHz, indicating good low-frequency polarization conversion potential. However, its reflection response deteriorates rapidly above 20 GHz, which limits the overall operating bandwidth.

To extend the bandwidth, we introduced additional inner metallic elements in Model-B. These elements are not used merely to increase structural complexity. Their main role is to provide extra current paths and local capacitive coupling inside the SRR, which helps excite additional resonant modes and improves the response continuity in the middle-frequency range. As shown in Figure 1b, Model-B gives a smoother response within 15–25 GHz, but the reflection coefficient still rises above −10 dB at some frequencies between 25 and 30 GHz.

Model-C further simplifies and regularizes the inner elements. Compared with the relatively irregular elements in Model-B, the two symmetric square patches in Model-C provide a clearer structure and are easier to fabricate and tune. Meanwhile, these patches introduce patch-related resonant modes in the middle- and high-frequency ranges. These modes interact with the intrinsic resonances of the outer SRR and jointly support a continuous broadband polarization conversion response. This design follows the general strategy used in SRR-based, multi-resonance, and nested-resonator polarization converters, where several adjacent resonant modes are introduced within a compact unit cell to broaden the operating bandwidth.

The optimized Model-C finally shows a more stable reflection response across 10–30 GHz and effectively extends the high-frequency operating range. Therefore, Model-C was chosen as the final unit-cell configuration in this work.

**Figure 2:**
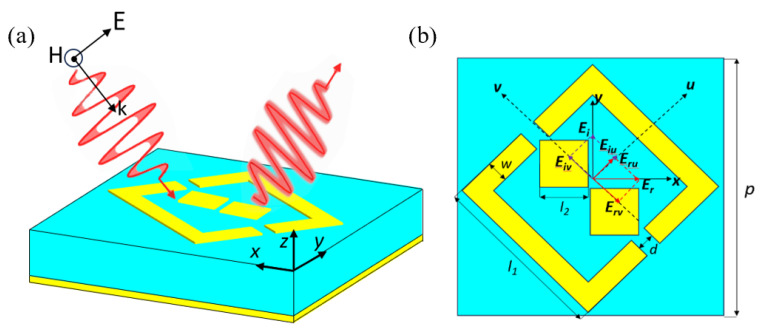
Schematic of polarization conversion. (**a**) Three-dimensional view. (**b**) Schematic diagram.

The proposed polarization-converting metasurface, as shown in Figure 2, converts incident plane waves of a given polarization into their cross-polarized counterparts upon reflection. The unit cell of the metasurface comprises a top anisotropic metallic structure, a dielectric substrate, and a grounded metallic layer. As shown in Figure 2a, the top metal structure is designed as a combination of a double-square structure and a square slit ring resonator. Figure 2b displays the optimized geometric parameters: unit period *p* = 6.35 mm, with other parameters being *d* = 0.3 mm, *l*_1_ = 4.3 mm, *l*_2_ = 0.63 mm, and *w* = 0.4 mm. Both the top and bottom metal structures consist of copper films with a thickness of 0.035 mm and a conductivity of σ = 5.8 × 10^7^ S/m. An F4B dielectric substrate with a thickness of 2 mm, a relative permittivity of 2.65, and a loss tangent of 0.001 was selected.

### 2.2. Simulation Results

**Figure 3:**
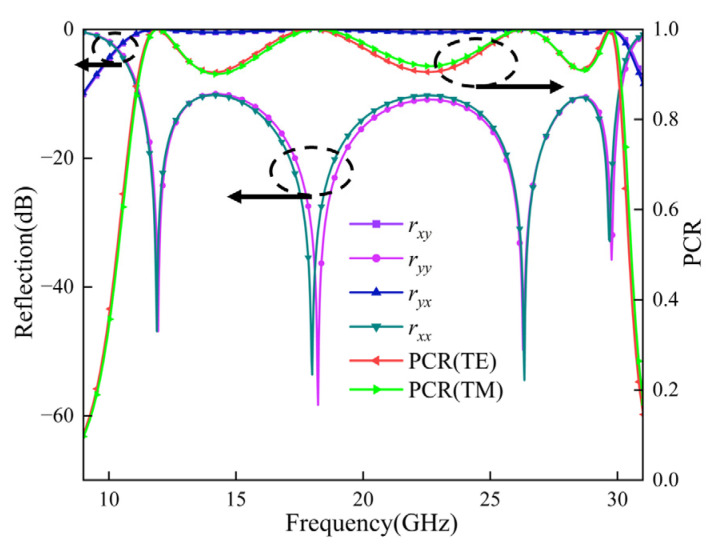
Co-polarized and cross-polarized reflection coefficients and PCR under normal incidence for both *x*- and *y*-polarized incidences.

To evaluate the polarization conversion performance of the proposed metasurface, we carried out full-wave simulations in CST Microwave Studio. We applied periodic boundary conditions along the *x*- and *y*-directions and used an open boundary along the *z*-direction. Figure 3 compares the co-polarized and cross-polarized reflection coefficients, together with the corresponding PCR, under *x*- and *y*-polarized normal incidences.

We first examine the *y*-polarized case in Figure 3. Across the frequency range from 10.89 to 30.12 GHz, the co-polarized reflection coefficient $r_{yy}$ stays below −10 dB, while the cross-polarized reflection coefficient $r_{xy}$ remains close to 0 dB. This result indicates that the metasurface can efficiently convert the incident wave into its orthogonal polarization over a broad band. We attribute this broadband response to four closely spaced resonances located at 11.91, 18.20, 26.29, and 29.77 GHz. Instead of forming several isolated narrow bands, these resonances couple with each other and produce a continuous polarization conversion band. To describe the conversion efficiency more clearly, we use the polarization conversion ratio (PCR), which is defined as $\;\mathrm{PCR}=r_{xy}^{2}/(r_{xy}^{2}+r_{yy}^{2})$, where $r_{xy}$ and $r_{yy}$ denote the cross-polarized and co-polarized reflection coefficients, respectively, under *y*-polarized normal incidence. A larger PCR corresponds to stronger polarization conversion. As shown by the red curve in Figure 3, the PCR remains higher than 0.9 throughout the 10.89–30.12 GHz band, which means that more than 90% of the incident energy is converted into the orthogonal polarization. Around the four resonant frequencies, the PCR approaches 1.

Figure 3 compares the co-polarized and cross-polarized reflection coefficients and PCR under *x*- and *y*-polarized normal incidences. For *y*-polarized incidence, $r_{xy}\;$ remains high over the operating band, while $r_{yy}\;$ stays low. For *x*-polarized incidence, $r_{yx}\;$ shows a similarly high response and $r_{xx}\;$ remains strongly suppressed. The two PCR curves are also very close, confirming that the proposed metasurface realizes efficient broadband cross-polarization conversion for both orthogonal linear polarizations.

The close amplitude responses mainly come from the near-symmetric in-plane arrangement of the unit cell and the reciprocal response under normal incidence. However, the split-ring gap and the local interaction between the inner square patches and the outer SRR break strict fourfold rotational symmetry. Therefore, *x*- and *y*-polarized incidences excite very similar but not exactly identical local current distributions and coupling strengths. This explains the slight differences between $r_{xx}\;$ and $r_{yy}$, as well as between the two PCR curves. Minor numerical factors in CST, such as mesh discretization, field interpolation, and the computational-grid orientation, may also contribute to these small deviations.

It should also be noted that similar amplitude responses under pure *x*- and *y*-polarized incidences do not guarantee the same PCR for arbitrary incident polarization angles. For a rotated linearly polarized incidence, the reflected *x*- and *y*-components recombine into the final reflected field, and this process depends on both their amplitudes and their relative phase. Therefore, Section 2.7 further discusses the influence of incident polarization angle.

### 2.3. Working Principle

**Figure 4:**
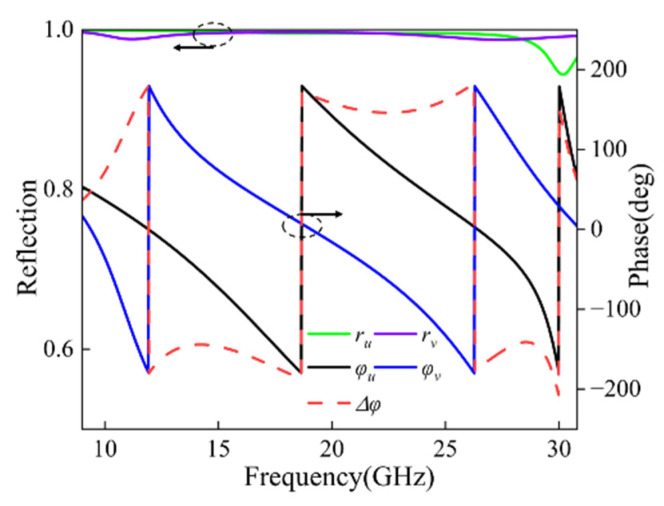
Reflected Amplitude and Phase Difference of $r_{u}$ and $r_{v}$.

To further understand the operating principle of the proposed metasurface, we define the *u*-axis and *v*-axis to analyze the structure’s anisotropy, as shown in Figure 2b. The *u*-axis and *v*-axis are obtained by rotating the *x*-*y* axes counterclockwise by 45°. When irradiated by a *y*-polarized wave, the incident electric field *Ei* decomposes into two orthogonal components along the *u*-axis and *v*-axis. If the conditions $\;\left|r_{u}\right|=\left|r_{v}\right|\;$ and $\;|\varphi_{u}-\varphi_{v}|\approx 180{}^{\circ}$ in [31] are satisfied, the reflected electric field *Er* will propagate along the *x*-direction. That is, the linearly polarized incident wave is twisted to an orthogonal polarization direction after reflection by the unit cell, signifying the completion of polarization conversion. Figure 4 presents the amplitude distributions of $r_{u}$ and $r_{v}$ along with their phase difference. It is evident that within the frequency range of 10.89 GHz to 30.12 GHz, the reflection amplitudes of $r_{u}$ and $r_{v}$ are approximately 1, while the phase difference consistently remains close to ±180°. These results strongly validate that the designed metasurface achieves highly efficient polarization conversion across the entire operating frequency range.

### 2.4. Physical Mechanism

To further reveal the physical mechanism of broadband polarization conversion, we analyzed the surface current distributions on the top metallic pattern and the bottom ground plane at four representative resonant frequencies, namely 11.91 GHz, 18.20 GHz, 26.29 GHz, and 29.77 GHz. By examining the current direction and distribution, the dominant resonance behavior at each frequency can be identified.

**Figure 5:**
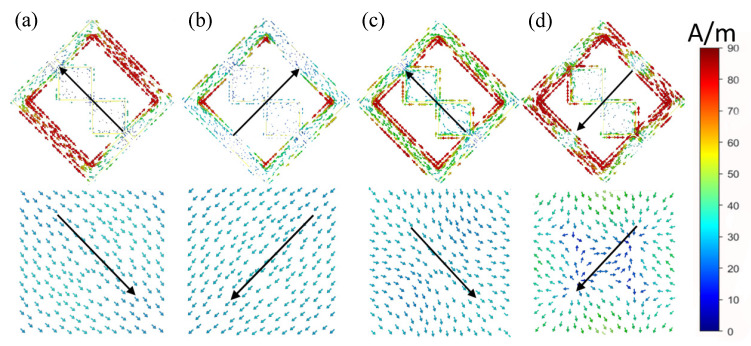
Surface current distribution of the resonator and metal ground at different resonance positions: (**a**) 11.91 GHz, (**b**) 18.20 GHz, (**c**) 26.29 GHz, and (**d**) 29.77 GHz.

As shown in Figure 5a, under *y*-polarized incidence at 11.91 GHz, the current on the top resonator is mainly distributed along the positive *v*-axis, while the current on the bottom ground plane flows along the negative *v*-axis. The currents are mainly concentrated on both sides of the resonator. Since the currents on the top layer and the ground plane are antiparallel, this resonance can be identified as a magnetic resonance. At 18.20 GHz, as shown in Figure 5b, the current is mainly concentrated at the four corners of the split-ring resonator. The current on the top metallic pattern flows in the opposite direction to that on the ground plane, again indicating a magnetic resonance. A similar phenomenon can be observed at 26.29 GHz in Figure 5c, where the current is mainly concentrated on the upper patch and remains opposite to that on the ground plane. Therefore, this resonance also corresponds to a magnetic mode.

The situation at 29.77 GHz is different from the previous three cases. As shown in Figure 5d, the top-layer current is distributed not only around the edges of the inner square patches, but also along the inclined arms and corners of the outer SRR. Unlike the lower-frequency resonances, where the top metallic layer and the ground plane mainly form clear antiparallel current distributions, the current at 29.77 GHz does not correspond to a simple global current loop. Instead, strong localized currents appear around both the inner patches and the outer SRR edges. This feature indicates that the resonance is neither an isolated inner-patch resonance nor a pure SRR magnetic resonance. The inner patches contribute localized electric-response-related current paths, while the outer SRR still participates in the resonance through electromagnetic coupling. Therefore, this resonance is more reasonably interpreted as a patch-assisted high-order hybrid electromagnetic mode formed by the interaction between the inner square patches and the outer SRR.

In addition, the induced current on the bottom ground plane no longer shows the simple global antiparallel mirror-like distribution observed in the lower-frequency magnetic resonances. Instead, it exhibits more localized perturbations near the central region and the projection area of the inner patches, further supporting the interpretation of a patch-assisted hybrid electromagnetic mode.

This high-frequency hybrid mode plays an important role in extending the operating bandwidth. It provides an additional high-frequency resonant channel, compensates for the weakening of the SRR-dominated response at the high-frequency end, and helps maintain strong cross-polarized reflection near 30 GHz.

Overall, the broadband polarization conversion of the proposed metasurface results from the combined contribution of several adjacent resonant modes, including the lower-frequency magnetic-type resonances and the high-frequency patch-assisted hybrid mode. The interaction among these modes jointly produces the continuous broadband polarization conversion over 10.89–30.12 GHz.

### 2.5. Parameter Analysis

We optimized the unit cell through a physically guided parametric sweep. During the tuning process, the outer SRR mainly controls the lower-frequency resonances, whereas the inner square patches adjust the middle- and high-frequency modes. We further tuned the strip width and split gap to balance the resonance depth, bandwidth continuity, and PCR level. For designs targeting other frequency bands, proportional scaling provides a useful initial estimate, but further refinement remains necessary because the material properties, metal thickness, substrate thickness, and patch–SRR coupling do not scale ideally.

**Figure 6:**
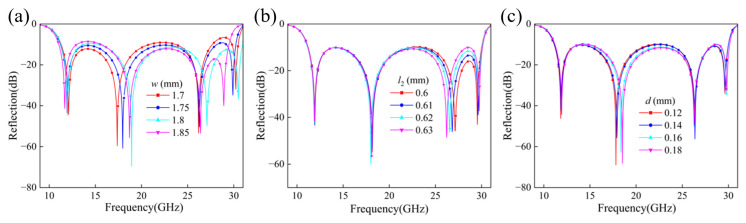
Co-polarization reflection coefficients for different geometric parameters: (**a**) Width *w*, (**b**) Length *l*_2_, and (**c**) Width *d*.

We further investigated the influence of structural geometric parameters on polarization conversion performance by performing parameter scans on three key dimensions: *w*, *l*_2_, and *d*. As parameter *w* increases, as shown in Figure 6a, the structure exhibits distinct resonances near 12 GHz, 18–19 GHz, 26 GHz, and 30 GHz, with resonance frequencies overall exhibiting a certain redshift trend. This shift reduces reflection stability at high frequencies, thereby affecting overall bandwidth and polarization conversion efficiency. From an electromagnetic perspective, this occurs because increasing *w* enlarges the metal edges and enhances the coupling between the patch and the SRR, effectively increasing the capacitance of the resonant elements and lowering their resonant frequencies.

As shown in Figure 6b, increasing *l*_2_ causes the third resonance frequency to shift toward lower frequencies, particularly evident between 18 GHz and 28 GHz. This indicates high sensitivity of polarization conversion performance and bandwidth to the *l*_2_ parameter within this frequency range, which can be attributed to the longer current path and larger patch area increasing the effective capacitance and inductance of patch-dominated modes.

Figure 6c indicates that parameter *d* has minimal impact on resonance at different frequencies. Resonance frequencies near 12 GHz and 26 GHz remain largely stable, with only slight variations in the minimum reflection coefficient. Near 18 GHz, however, increasing *d* causes a noticeable frequency shift in the resonance valley, accompanied by changes in the −10 dB bandwidth. Near 30 GHz, the resonance characteristics of all curves exhibit greater consistency, indicating that variations in *d* have a weaker influence on polarization conversion efficiency at higher frequencies. In summary, alterations in the geometric parameters *w*, *l*_2_, and *d* modulate the structure’s resonance frequency and bandwidth characteristics, thereby affecting its polarization conversion performance.

### 2.6. Angular Stability

**Figure 7:**
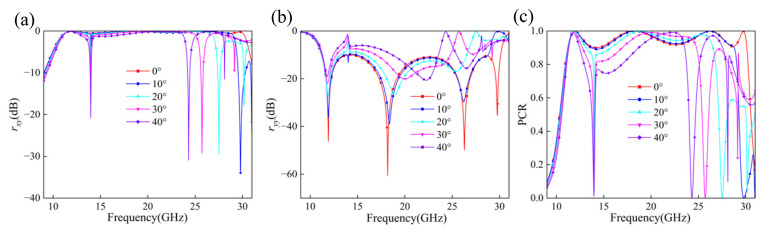
Reflection coefficients under oblique incidence: (**a**) $r_{xy}$, (**b**) $r_{yy}$, and (**c**) PCR.

We also investigated the polarization conversion characteristics of this metasurface under different incident angles. As shown in Figure 7a, the cross-polarization components exhibit an overall attenuation trend as the incident angle increases, which becomes particularly pronounced in the high-frequency region above 18 GHz. When the incident angle reaches 40°, the cross-polarization amplitude in the high-frequency band decreases significantly, indicating that the ability of the structure to excite and sustain cross-polarized responses is strongly degraded at large oblique incidences. Figure 7b reveals two distinct reflection troughs near approximately 13 GHz and 22 GHz, corresponding to the dominant resonant modes of the structure. As the incident angle increases from 0° to 40°, both resonance points exhibit a certain degree of blue shift, accompanied by a gradual shallowing of the reflection troughs. This behavior indicates that oblique incidence modifies the effective resonance conditions of the unit cells, alters the field coupling within the resonant elements, and weakens the resonance strength.

The polarization conversion rate (PCR) as a function of frequency is presented in Figure 7c. The results show that the proposed metasurface still maintains a high PCR over a large portion of the operating band under oblique incidence. In particular, when the incident angle is within 10°, the PCR remains high across almost the entire operating band. For larger incident angles of 20–40°, the PCR still stays at a relatively high level over many frequency regions, but a pronounced narrow-band degradation appears near 13–14 GHz. Therefore, the angular response should not be described as uniformly insensitive over the entire 11–14 GHz range. Instead, the proposed metasurface exhibits acceptable angular stability over most of the operating band, with localized angle-sensitive degradation near 13–14 GHz and at some high-frequency points.

The localized PCR drop near 13–14 GHz can be attributed to the angle-induced change in the resonant excitation condition. As the incident angle increases, the in-plane wavevector component becomes larger, and the incident electric field has a different projection on the anisotropic resonator. This changes the interaction between the outer SRR and the inner-patch-related mode and disturbs the amplitude balance and phase difference between the two orthogonal reflected components. As a result, the co-polarized reflection increases locally, while the cross-polarized conversion weakens, leading to the narrow PCR drop near 13–14 GHz. At higher frequencies, some higher-order and more localized resonant modes are also sensitive to oblique incidence, which explains the additional narrow dips observed at large incident angles.

### 2.7. Influence of Incident Polarization Angle

**Figure 8:**
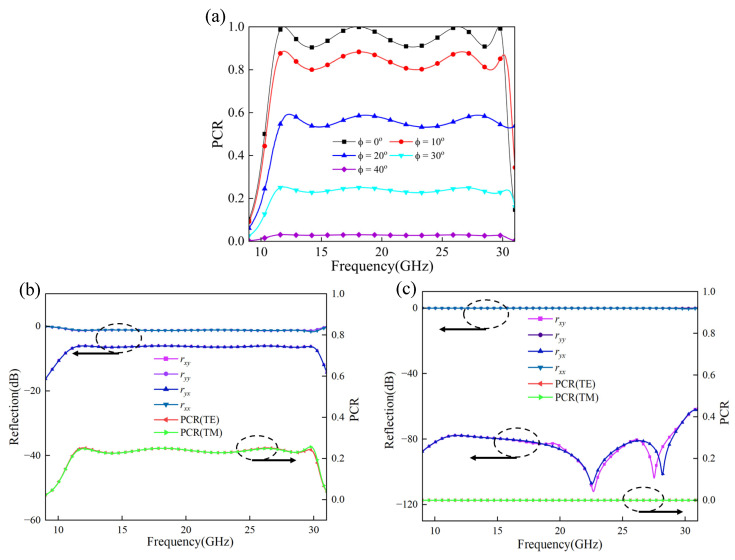
Influence of incident polarization angle on polarization conversion performance: (**a**) PCR curves; (**b**) responses at α = 30°; (**c**) responses at α = 45°.

Figure 8a shows the simulated PCR curves under different incident polarization angles. The PCR decreases as the incident polarization angle moves away from the principal *x*- and *y*-polarized directions. This behavior does not contradict the high conversion efficiency obtained under pure *x*- and *y*-polarized incidences in Figure 3.

For pure *x*- or *y*-polarized incidence, the metasurface mainly performs cross-polarization conversion between the two principal components. However, this does not mean that the structure works as a universal 90° polarization rotator for any incident linear polarization. When the incident polarization angle changes, the incident field can still be decomposed into *x*- and *y*-polarized components, but the final reflected polarization state depends on how the converted components recombine. This recombination is governed not only by the magnitudes of the two reflected components, but also by their phase relation.

To further check whether the PCR degradation comes from magnitude mismatch, Figure 8b and Figure 8c compare the reflection responses at incident polarization angles of 30° and 45°, respectively. The *x*- and *y*-related reflection curves remain very close at both angles, while the PCR still decreases clearly as the incident polarization angle increases. This indicates that the degradation does not mainly come from an amplitude mismatch between the two principal polarization responses.

Therefore, even though the *x*- and *y*-polarized responses are very similar under normal incidence, the reflected components may not always recombine along the direction exactly orthogonal to the incident polarization. When the phase relation between the reflected components does not satisfy the ideal condition for arbitrary-angle 90° rotation, part of the reflected field projects onto the non-target polarization direction, leading to a reduced PCR. Thus, the polarization-angle-dependent PCR degradation is mainly associated with the phase-related vector recombination of the reflected components rather than numerical error or simple amplitude mismatch.

### 2.8. RCS Reduction and Far-Field Scattering Analysis

**Figure 9:**
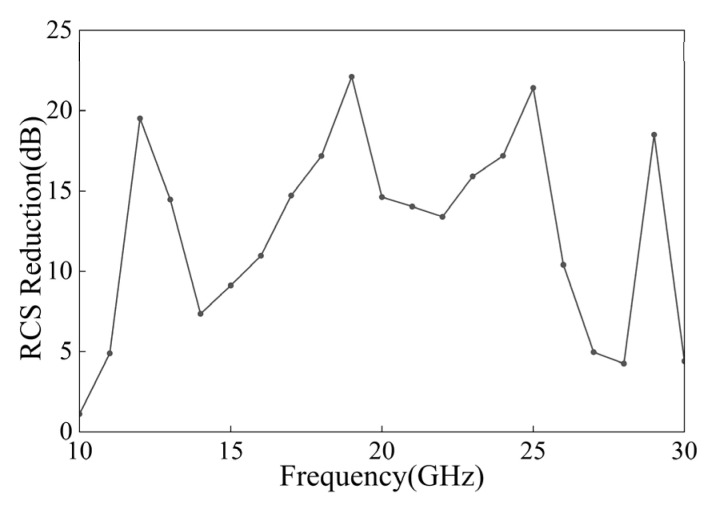
Monostatic RCS reduction in the proposed metasurface.

In addition to broadband polarization conversion, the proposed metasurface also shows scattering characteristics relevant to radar scattering suppression. Here, we use the RCS results as a simulation-based supplementary analysis to indicate the potential scattering-suppression application of the proposed metasurface, rather than as a fully experimentally verified RCS performance. To examine this application’s potential, we carried out full-wave simulations of both its monostatic RCS reduction and three-dimensional far-field scattering behavior. Specifically, we constructed a 5 × 5 checkerboard metasurface array and compared its monostatic RCS with that of an identically sized perfect electric conductor (PEC) plate. Figure 9 shows that the proposed metasurface has the potential to achieve pronounced monostatic RCS reduction over a wide frequency range. In particular, the reduction exceeds 20 dB at several representative frequencies. These RCS reduction peaks appear near the resonance frequencies where strong cross-polarization conversion occurs, suggesting a close relationship between polarization conversion and backscattering suppression.

**Figure 10:**
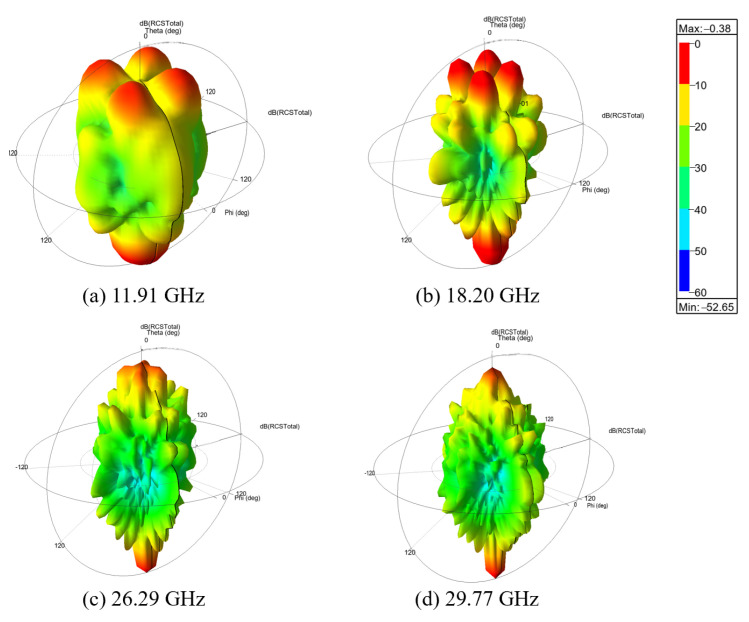
Three-dimensional far-field scattering patterns at different resonant frequencies.

To gain further insight into the scattering behavior, Figure 10a–d presents the three-dimensional far-field scattering patterns at four representative resonant frequencies, namely 11.91 GHz, 18.20 GHz, 26.29 GHz, and 29.77 GHz. Unlike the PEC plate, which mainly produces strong specular reflection in the normal direction, the proposed checkerboard metasurface redistributes the scattered energy into several off-normal directions. Consequently, the backscattered energy along the normal direction is effectively reduced.

These simulation results suggest that the proposed metasurface may provide potential for radar scattering suppression and electromagnetic stealth-related applications, in addition to broadband polarization conversion. Further experimental RCS validation will require a dedicated calibrated measurement system.

## 3. Experimental Validation and Discussion

**Figure 11:**
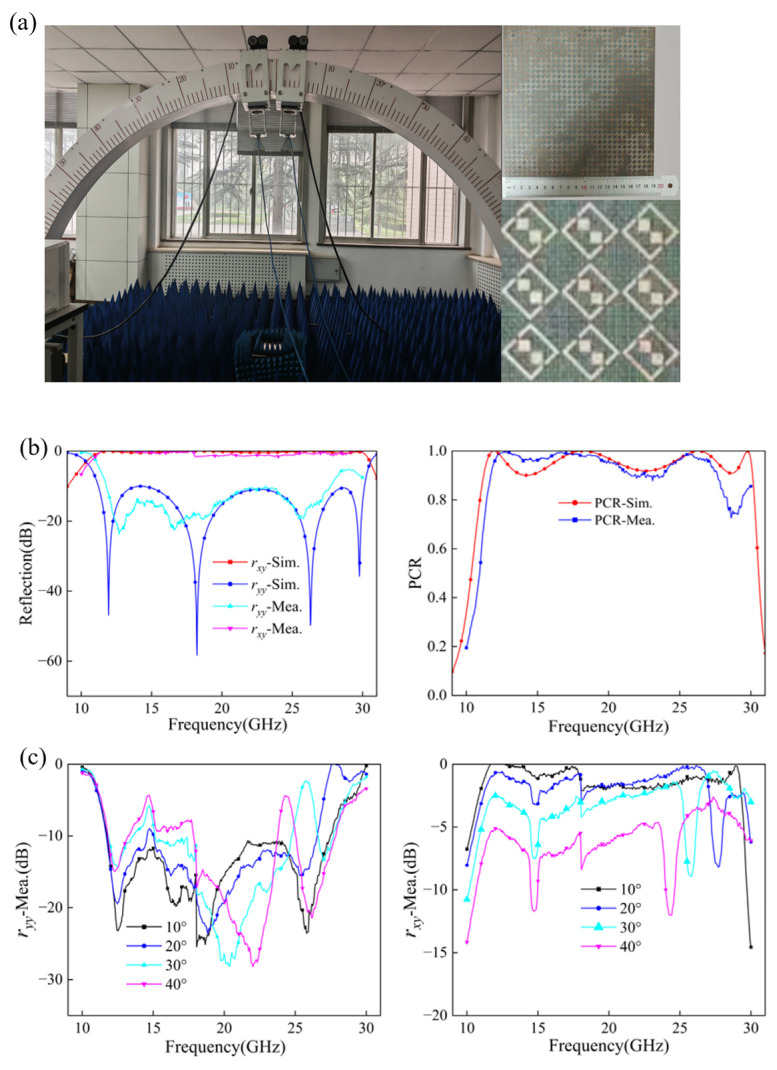
(**a**) Measurement setup and prototype fabrication. (**b**) Simulation and measurement results for $r_{yy}$, $r_{xy}$, and PCR. (**c**) Measurement results for $r_{yy}$ and $r_{xy}$ under oblique incidence.

We fabricated the proposed metasurface sample and experimentally verified its performance. As shown in Figure 11a, the metasurface structure consists of a 30 × 30 unit cell array with a total size of 190.5 × 190.5 mm^2^. The measurement system comprised two standard gain horn antennas connected to a vector network analyzer (Keysight PNA-L Network Analyzer N5234B, Keysight Technologies, Santa Rosa, CA, USA). One horn antenna irradiated the metasurface with y-polarized EM (electromagnetic) waves, while the other received *y*- or *x*-polarized reflected waves to obtain the co-polarized and cross-polarized reflection coefficients $r_{yy}$ and $r_{xy}$. The sample was placed on a platform in front of the horn antenna and surrounded by absorptive material to mitigate electromagnetic interference. Figure 11b shows a comparison of measured reflection coefficients and PCR values with simulation results, demonstrating good agreement between experimental and theoretical data. Figure 11c further presents the measured polarization conversion performance under oblique incidence. It can be observed that the measured results follow the same trend as the simulated angular responses shown in Figure 7a,b, further supporting the acceptable oblique-incidence performance of the proposed metasurface over a large portion of the operating band.

Some differences between the simulated and measured results still remain, especially in the high-frequency region. We attribute these deviations to several practical factors. First, we carried out the measurements in a laboratory setup rather than in a fully anechoic chamber. Although we placed absorbers around the sample to suppress unwanted reflections, scattering from surrounding objects could not be completely eliminated. At high frequencies, where the wavelength becomes much shorter, the measured response becomes more sensitive to such environmental multipath interference, which introduces fluctuations into the curves.

We also introduced additional uncertainty in the measurement process itself. To cover the full 10–30 GHz operating band, we used two horn antennas working in different frequency ranges (9–18 GHz and 18–40 GHz). During the experiment, we had to replace the antennas and reconnect the RF (radio frequency) cables, and these operations inevitably introduced slight alignment errors and phase-calibration deviations. In addition, the finite size of the fabricated sample and the associated edge diffraction, which are not considered in the infinite-periodic simulations, also contribute to the mismatch between simulation and measurement. These factors influence not only the simulated–measured comparison in Figure 11b, but also the measured oblique-incidence results shown in Figure 11c.

At the same time, the metasurface itself becomes more sensitive in the high-frequency band. In the 28–30 GHz range, the operating wavelength approaches the characteristic dimensions of the unit cell, so small fabrication errors have a more noticeable effect on the electromagnetic response. Slight dimensional deviations in the square patches and split-ring resonators can change the local parasitic capacitance and inductance, which then shift the resonant frequencies and lower the polarization conversion efficiency. In addition, the dielectric loss of the substrate usually increases with frequency under practical conditions, and this further reduces the measured performance compared with the ideal simulations. Taken together, these factors provide a reasonable explanation for the remaining discrepancies, while the overall agreement between simulation and experiment remains satisfactory.

Table 1 compares the proposed metasurface with several previously reported polarization-converting metasurfaces. Besides operating bandwidth, relative bandwidth, thickness, and the number of dielectric layers, we also include the average PCR at θ = 30° so that the oblique-incidence performance can be compared more directly. As the table shows, some reported designs achieve better angular stability, but they usually rely on multilayer structures and therefore come with increased thickness and fabrication complexity. By contrast, the proposed design keeps a compact single-layer passive configuration and still maintains acceptable performance under oblique incidence. Overall, it provides a reasonable balance among bandwidth, conversion efficiency, angular stability, and structural simplicity.

It should also be noted that the proposed design does not exhibit the highest relative bandwidth among all compared works. For example, the work listed as Ref. [27] shows a larger relative bandwidth. However, that design employs a two-layer dielectric configuration and has a larger normalized thickness. In contrast, the proposed metasurface achieves continuous broadband polarization conversion using a single-layer passive structure with a lower normalized thickness of 0.071**λ_max_**. Moreover, the proposed structure does not require complicated multilayer alignment or post-assembly processes, making it more compatible with standard PCB fabrication. Therefore, the main advantage of the proposed design lies in its balanced performance among broadband conversion, low profile, structural simplicity, and fabrication feasibility.

## 4. Summary

In this paper, we designed a linear polarization-converting metasurface with a single-layer and low-profile structure. The proposed metasurface maintains a polarization conversion ratio above 90% over 10.89–30.12 GHz, showing a wide operating band together with a compact configuration. Our analyses of the phase response, surface current distributions, and structural parameters indicate that the broadband response originates from the interaction of several adjacent resonant modes. In particular, the high-frequency patch-assisted hybrid mode helps extend the effective conversion band toward 30 GHz. The structure also maintains acceptable performance under small oblique incidence, although the high-frequency response becomes more sensitive at larger incident angles. The measured results agree well with the simulations, which supports the effectiveness of the design. In addition, the simulation-based scattering analysis suggests that this metasurface may provide potential for radar scattering suppression, while dedicated calibrated RCS measurements will be considered in future work. Because the structure is simple and easy to fabricate, it could be a practical option for microwave systems that require broadband polarization conversion.

## Figures and Tables

**Table 1:** Comparison with Other Polarization-Converting Metasurfaces.

Works	Operating Bandwidth (GHz)	Bandwidth (GHz)	Relative Bandwidth (PCR > 0.9) (%)	Thickness (h/λ_max_)	Dielectric Layers	Angular Stability (Average PCR at *θ* = 30°)
[7]	6.67–17.09	10.42	87.7	0.078	1	N/A
[21]	5.71–15.02	9.31	89.8	0.076	3	N/A
[27]	6.30–20.50	14.2	105.9	0.084	2	≈90%
[8]	5.69–14.82	9.13	89	0.076	1	≈85%
[12]	9.50–18.50	9	64.3	0.084	1	≈80%
Our work	10.89–30.12	19.23	94	0.071	1	≈85%

## Data Availability

The data supporting the findings of this study are included in the article. Additional simulation and measurement data are available from the corresponding authors upon reasonable request.
